# All-Optically Controlled Memristive Device Based on Cu_2_O/TiO_2_ Heterostructure Toward Neuromorphic Visual System

**DOI:** 10.34133/research.0580

**Published:** 2025-01-10

**Authors:** Jun Xie, Xuanyu Shan, Ningbo Zou, Ya Lin, Zhongqiang Wang, Ye Tao, Xiaoning Zhao, Haiyang Xu, Yichun Liu

**Affiliations:** Key Laboratory for UV Light-Emitting Materials and Technology (Ministry of Education), College of Physics, Northeast Normal University, Changchun, China.

## Abstract

The optoelectronic memristor integrates the multifunctionalities of image sensing, storage, and processing, which has been considered as the leading candidate to construct novel neuromorphic visual system. In particular, memristive materials with all-optical modulation and complementary metal oxide semiconductor (CMOS) compatibility are highly desired for energy-efficient image perception. As a p-type oxide material, Cu_2_O exhibits outstanding theoretical photoelectric conversion efficiency and broadband photoresponse. In this work, an all-optically controlled memristor based on the Cu_2_O/TiO_2_/sodium alginate nanocomposite film is developed. Optical potentiation and depression behaviors have been implemented by utilizing visible (680 nm) and ultraviolet (350 nm) light. Furthermore, a 7 × 9 optoelectronic memristive array with satisfactory device variation and environment stability is constructed to emulate the image preprocessing function in biological retina. The random noise can be reduced effectively by utilizing bidirectional optical input. Beneficial from the image preprocessing function, the accuracy of handwritten digit classification increases more than 60%. Our work presents a pathway toward high-efficient neuromorphic visual system and promotes the development of artificial intelligence technology.

## Introduction

The conventional machine vision is composed of image sensing, storage, and processing units, which features a physical separation from each other [1]. Thereinto, the analog-to-digital conversion and frequent data transmission between these components result in elevated speed latency and energy consumption [2–4]. In contrast, the human visual system (HVS) enables to process enormous image information simultaneously with high speed and low energy consumption, relying on sophisticated neural networks [5–7]. Inspired by the HVS, developing neuromorphic hardware with in-sensor computing architecture would provide an ideal platform for high-efficient image perception [8–11].

Optoelectronic memristor, as an emerging neuromorphic device, has been considered as promising candidate to construct neuromorphic visual system, due to the functional and structural resemblances to biological optical synapses [12–14]. In particular, several neuromorphic visual functions have been demonstrated in the field, including motion detection [15–18], pattern recognition [19–21], and image encryption [22]. In these results, the hybrid optical-electrical operations are usually indispensable to achieve reversible modulation of device conductance, which is unfavorable for real-time image processing with high efficiency [23]. Therefore, developing novel all-optical controlled memristor represents an alternative strategy to eliminate the hardware redundancy and high-power consumption in complicated optical-electrical operation [24–27]. Furthermore, the reversible modulation characteristics in optical manner established a promising paradigm to emulate the antagonism shunting function of bipolar cells in human retina [28]. On this basis, the image preprocessing function can be implemented by using the all-optical modulation characteristics, which is beneficial to enhance imaging quality and accelerate subsequent processing. For example, Li et al. [29] achieved gate-tunable positive/negative photoconductivity in PtS_2_/hBN/graphene floating gate memory and demonstrated the high-precision data classification. However, the current physical models and memristive materials for all-optically controlled synaptic devices are still quite limited. Therefore, exploring novel all-optically controlled memristor is highly desired for high-efficient neuromorphic visual perception.

Heterostructures with designable electronic interfaces enables to integrate various material advantages for high-performance optoelectronic behaviors and eliminate the complicated hardware circuits [30–34]. Herein, efficient separation of photogenerated electron-hole pairs and excellent light absorbance can be realized in the heterojunction structure [35–38]. In particular, zero-dimensional materials with ultra-high specific surface area provide abundant contact sites for efficient charge transfer at heterogeneous interface [39–43]. At present, constructing zero-dimensional heterojunction is the general strategy for efficient photocatalysis [44]. Inspired by these advances, the all zero-dimensional heterojunction provides a reference model for all-optically controlled memristor. As an intrinsic p-type semiconductor oxide, Cu_2_O exhibits a bandgap width of 2.0 eV, ensuring the excellent light absorbance in the ultraviolet (UV)-visible region [45]. On the other side, TiO_2_ with a wide band gap of ~3.2 eV is regarded as a leading candidate for future optoelectronic device, due to the outstanding properties of chemical stability and high carrier mobility [46–48]. As above, the Cu_2_O/TiO_2_ zero-dimensional heterojunction provides an excellent foundation for all-optical modulated memristor. However, to the best of our knowledge, the all-optically controlled memristor based on Cu_2_O/ TiO_2_ heterojunction has not been reported.

In this work, we demonstrate an all-optically controlled memristor based on Cu_2_O/TiO_2_ heterojunction, in which the conductance can be reversibly modulated by utilizing visible (680 nm) and UV (350 nm) light. Based on the optical potentiation/depression behaviors, versatile synaptic functions have been emulated, including excitatory/inhibitory post-synaptic currents (EPSP/IPSP), paired-pulse facilitation/depression (PPF/PPD), and long-term plasticity (LTP/LTD). Furthermore, image preprocessing function has been implemented to enhance feature information and suppress random noise. Then, we constructed a single-layer artificial neural network and demonstrated the classification of handwritten digits, showing recognition accuracy exceeding 90%. The proposed retinomorphic device provides a feasible strategy to develop high-efficient neuromorphic visual system.

## Results

**Fig. 1.**
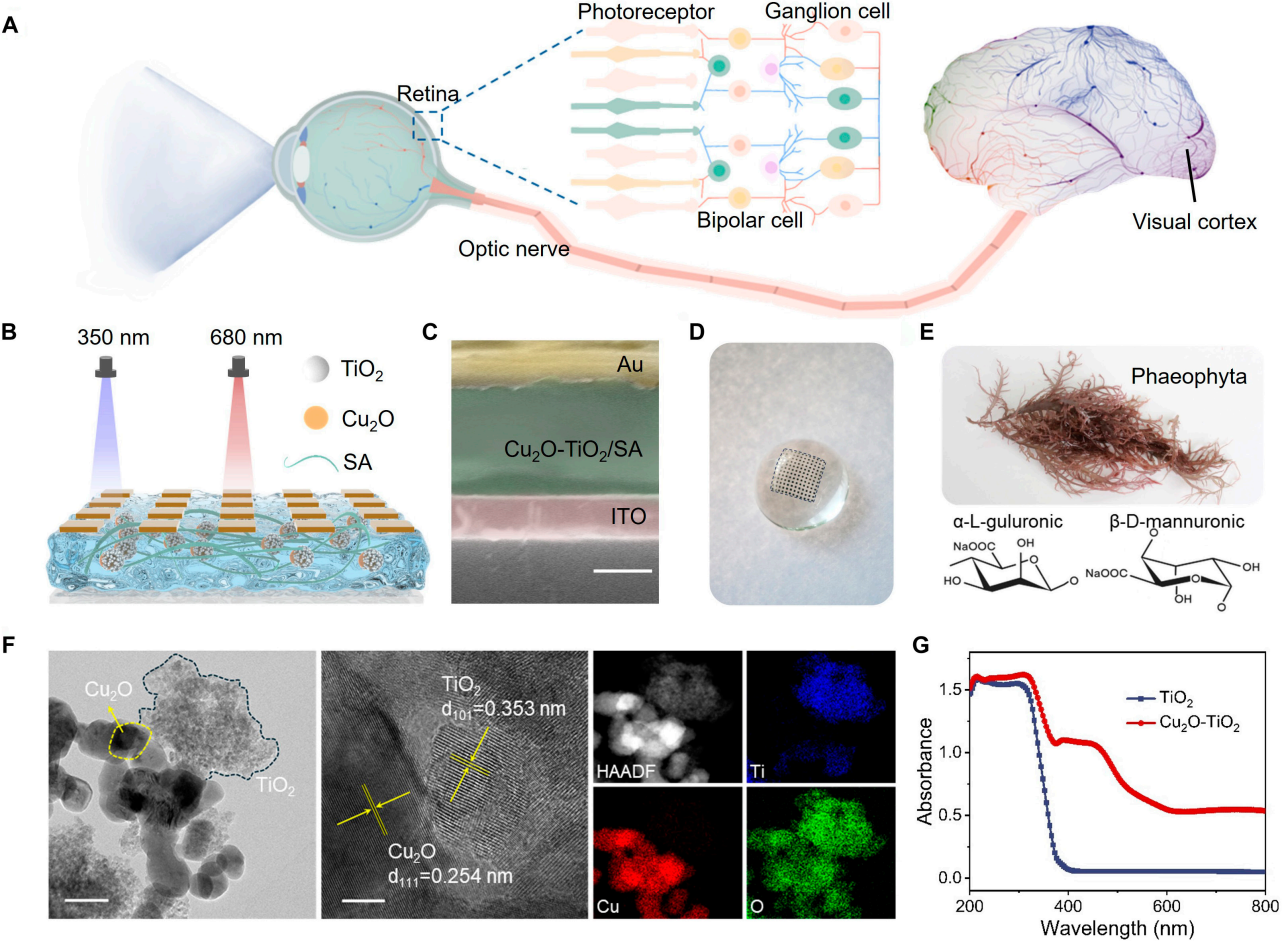
All-optically controlled memristor for neuromorphic vision. (A) Schematic diagram of HVS. (B and C) Schematic and cross-sectional scanning electron microscopy images of Cu_2_O–TiO_2_/sodium alginate-based optoelectronic memristor. Scale bar, 250 nm. (D) Photograph of optoelectronic memristive array attached on hemispherical substrate. (E) Source and molecular structure of sodium alginate. (F) High-resolution TEM image of Cu_2_O–TiO_2_ nanoparticles (scale bars, 50 and 5 nm). (G) Absorption spectra of Cu_2_O–TiO_2_ and TiO_2_ nanoparticles.

Humans obtain more than 80% of external information through the visual system, which is the most essential sensory organs to perceiving their surroundings. Figure 1A illustrates the motivation for developing multifunctional optoelectronic memristor to emulate biological visual system [49]. As depicted in the schematic diagram of HVS, the photoreceptors in biological retina convert the light signals into electrical signals. Meanwhile, the preprocessing capabilities in retina enable to reduce image noise and filter out redundant information. Then, image information is transmitted to the visual cortex through optic nerve for high-level processing and memorization [50]. In order to emulate the human retina, we developed a TiO_2_/Cu_2_O heterojunction memristor with all-optical modulation characteristics. As shown in Fig. 1B, the nanocomposite film consisting of Cu_2_O–TiO_2_ nanoparticles and sodium alginate acts as functional layer. Herein, the Cu_2_O–TiO_2_ nanoparticles acts as functional unit for optoelectronic memristive behaviors. Meanwhile, the sodium alginate is the base materials for desirable mechanical flexibility. The cross-sectional scanning electron microscopy image indicates that the thickness of the Cu_2_O–TiO_2_ film is ~490 nm (Fig. 1C). The root mean square roughness values of the nanocomposite film is 2.61 nm, indicating excellent film smoothness and uniformity (Fig. S1). The optoelectronic memristive array can be attached to the hemispherical substrate without obvious bubbles or wrinkles, indicating a conformal contact (Fig. 1D). It is worth noting that the low temperature is necessary for the device fabrication process due to the flexible substrates [51]. The above nonplanar structure is beneficial to achieve a wide field of view. Meanwhile, the natural polysaccharide material (sodium alginate) contains abundant hydrophilic functional groups, which enables a simple solution fabrication of the nanocomposite film (Fig. 1E and Fig. S2) [52]. Figure 1F shows the transmission electron microscopy (TEM) image of TiO_2_ and Cu_2_O nanoparticles. Herein, the average diameters of TiO_2_ and Cu_2_O are 6.3 and 39.2 nm, respectively (Fig. S3). The lattice fringes with spacings of 0.254 nm and 0.353 nm correspond to (111) plane of cubic Cu_2_O and (101) plane of anatase TiO_2_ [53,54]. The above result has been further confirmed in the selected-area electron diffraction (SAED) and x-ray diffraction (XRD) pattern analyses (Fig. S4). The energy-dispersive spectroscopy indicates that the 2 oxide nanoparticles combined with each other, indicating the formation of zero-dimensional heterojunction. The UV-visible absorption spectra of TiO_2_ and Cu_2_O/TiO_2_ nanoparticles are depicted in Fig. 1G. It can be seen that TiO_2_ nanoparticles exhibit steep absorption in the UV region, corresponding to the TiO_2_ bandgap of 3.2 eV. In contrast, a broad optical absorption from UV to visible range can be observed in the Cu_2_O–TiO_2_ sample, which can be attributed to the narrow bandgap (2.0 eV) of Cu_2_O nanoparticles.

**Fig. 2.**
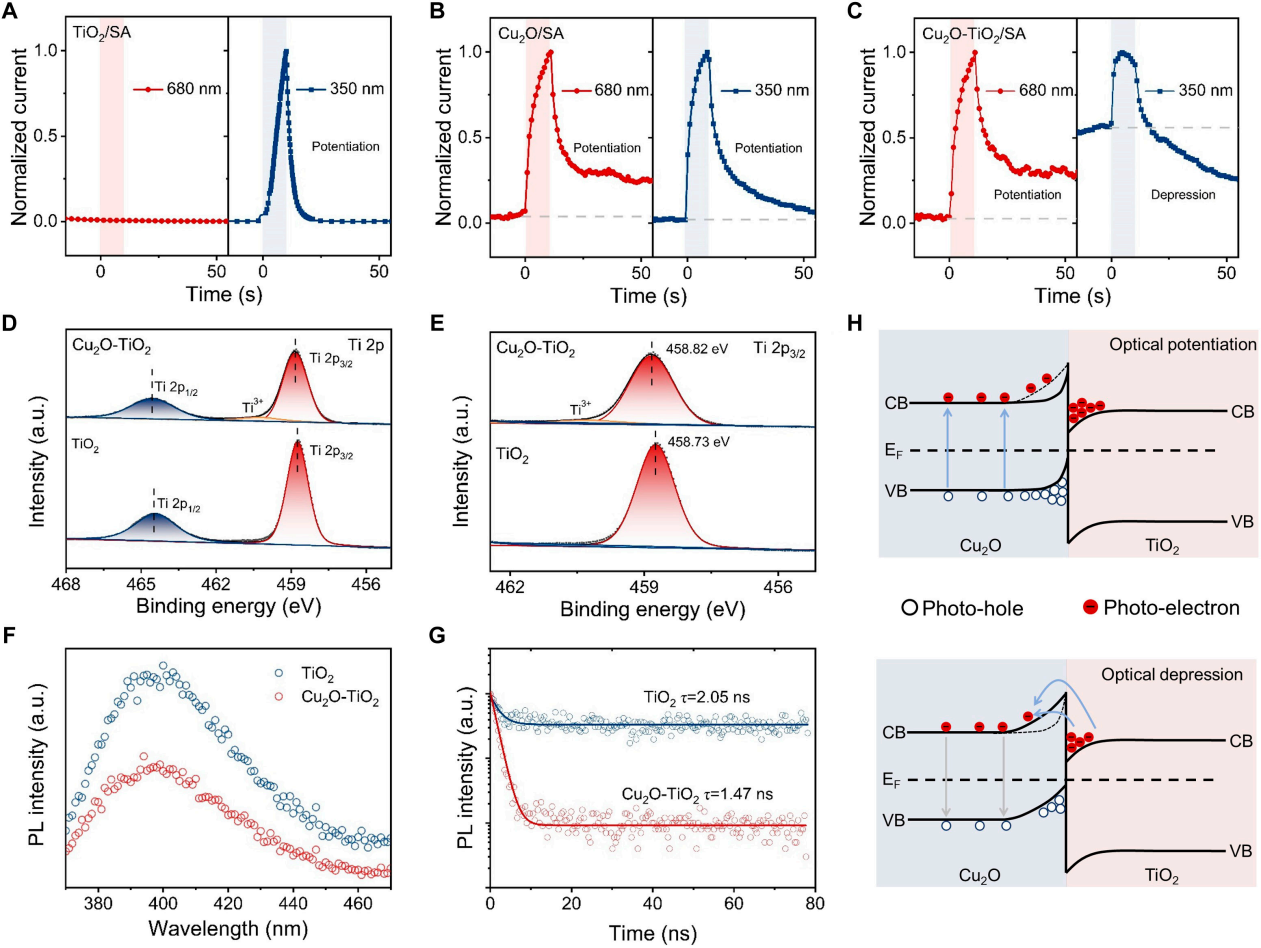
Operation mechanism for the all-optically controlled memristor. (A to C) Photoresponse behaviors of TiO_2_-, Cu_2_O-, and Cu_2_O–TiO_2_-based devices under visible (680 nm) and UV (350 nm) irradiation. (D and E) XPS spectra for Ti 2p in TiO_2_ and Cu_2_O–TiO_2_. (F and G) PL spectra and PL decay traces of TiO_2_ and Cu_2_O–TiO_2_. (H) Memristive mechanism of all-optical modulation characteristics.

The optimization of material component and mechanism investigation are first performed in our Cu_2_O/TiO_2_ heterojunction device. As shown in Fig. 2A, the TiO_2_/sodium alginate device exhibits optical potentiation behaviors under the action of UV light (350 nm). Meanwhile, the optical depression behavior is absent with the illumination of visible light (680 nm). For the Cu_2_O/sodium alginate device, optical potentiation behaviors are observed under UV/visible light, due to the narrow bandgap, as shown in Fig. 2B. In contrast, both optical potentiation and depression response behaviors have been obtained in the Cu_2_O/TiO_2_ heterostructure device, i.e., all-optical modulation characteristics. As shown in Fig. 2C, the device exhibits a transient potentiation current under the irradiation of UV light. After the UV illumination is removed, the heterojunction device shows a stable conductance state lower than the initial state, i.e., optical depression behaviors. On the other side, visible light induces obvious enhancement in transient current, which can be partly maintained after the optical signal is removed. Furthermore, the heterojunction device exhibits a broad spectrum of UV-visible response, as plotted in Fig. S5. As the light wavelength increases from 350 to 680 nm, the photoresponse behaviors gradually switch from depression to potentiation. The optical signals of 680 and 350 nm are selected for subsequent investigation, due to the remarkable photo-induced enhancement/inhibition effect. In order to investigate the underlying mechanism, x-ray photoelectron spectroscopy (XPS) analysis is conducted. Compared with the pure TiO_2_ sample, both the Ti 2p_1/2_ and Ti 2p_3/2_ redshifted for 0.09 eV in Cu_2_O/TiO_2_ (Fig. 2D and E) [55]. The redshifted peak can be attributed to the electron transfer between Cu_2_O and TiO_2_, which confirms the formation of Cu_2_O/TiO_2_ heterojunction. Besides that, photoluminescence (PL) emission spectra show that the emission intensity of Cu_2_O/TiO_2_ is significantly lower than pure TiO_2_ [56]. The transient PL spectra in Fig. 2F and G also show shorter lifetime in the Cu_2_O–TiO_2_ sample. The above results indicate the suppressed recombination of photogenerated electron-hole pairs in the oxide heterojunction. On this basis, a general model is proposed to explain the all-optically controlled behaviors, as illustrated in Fig. 2H. Herein, Cu_2_O/TiO_2_ heterojunction with type-II band alignment is formed (Fig. S6). When the Cu_2_O–TiO_2_-based device is irradiated with visible light (680 nm), the photogenerated electron-hole pairs in Cu_2_O nanoparticles decrease the barrier width, resulting in the conductance increase. For TiO_2_ nanoparticles, the electrons in conduction band cannot be excited under visible irradiation, due to the bandgap of 3.2 eV. After the visible light is removed, part of photogenerated electrons will recombine with holes in Cu_2_O, which corresponds to spontaneous decay in photocurrent. On the other side, UV light induces photogenerated electron-hole pair in both Cu_2_O and TiO_2_, resulting in transient potentiation behaviors. When the UV irradiation is removed, the photogenerated electrons in the potential well efficiently recombine with holes generated from Cu_2_O segment by tunnel through or jump over the interface barrier. The above process will increase the barrier width. Hence, the device exhibits optical depression behaviors under the action of UV light.

**Fig. 3.**
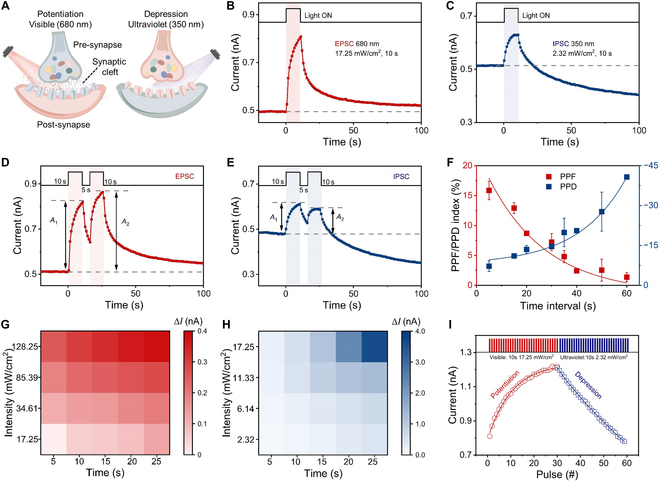
All-optical modulation characteristics in Cu_2_O–TiO_2_-based device. (A) Schematic diagram of all-optically controlled artificial synapse. (B and C) Synaptic EPSC and IPSC (inhibitory postsynaptic current) behaviors under the irradiation of visible (680 nm, 17.25 W/cm^2^, 10 s) and UV (350 nm, 2.32 mW/cm^2^, 10 s) light. (D and E) EPSC and IPSC behaviors in response to the pair of optical spikes (interval time of 5 s). *A*_1_ and *A*_2_ represent the photocurrent change at the first and second spike, respectively. (F) PPF and PPD index dependent on the time interval. (G and H) Photocurrent change as the function of irradiation duration (from 5 to 25 s) and intensity. (I) Reversible modulation under the action of alternate visible and UV spikes.

All-optically controlled synaptic plasticity is performed to emulate the photoresponse characteristics of bipolar cells in biological retina (Fig. 3A). Herein, the device current is regarded as synaptic weight, which is monitored with a bias voltage of 0.03 V. The optimization of film thickness is depicted in Fig. S7. Figure 3B depicts the photoresponse current response of the heterojunction device under visible light (17.25 mW/cm^2^, 10 s). Transient current enhancement of ~0.3 nA can be observed, which can be maintained for more than 100 s. The optical potentiation behavior is similar to the excitatory postsynaptic current (EPSC) in biological synapse. The ultra-low photocurrent of our all-optically controlled device is beneficial to reduce power consumption (see more details in Table S1). The device exhibits ultralow power consumption of 0.78 pJ, when the illumination duration reduces to 100 ms (Fig. S8). Furthermore, stable optoelectronic memristive behaviors can be achieved even after placing the device in atmosphere environment for ~300 d (Fig. S9). In contrast, Fig. 3C shows the photoresponse behaviors of our Cu_2_O–TiO_2_-based device under UV irradiation (2.32 mW/cm^2^, 10 s). After spontaneous relaxation, the stable state shows a lower device current than the initial state, indicating a long-term depression behavior. Furthermore, PPF and PPD have been demonstrated by utilizing 2 consecutive spikes, which are the foundation to decode temporary information. Figure 3D and E shows response curves under the stimulation of 2 consecutive visible and UV spikes (internal of 5 s). The photoresponse current induced by the second spike (*A*_2_) is obviously larger/smaller than that by the first one (*A*_1_). The facilitation and depression effects are similar with the PPF and PPD function in biological synapse. The PPF/PPD index can be calculated with the equation below: PPF/PPD index = (*A*_2_ − *A*_1_)/*A*_1_ × 100%. Herein, the time interval is the dominant factor for the PPF/PPD index. As the interval time increases from 5 to 60 s, the PPF index decreases from 15.87% to 1.37% and the PPD index decreases from –7.20% to –40.67% (Fig. 3F). The above indexes dependent on interval time can be fitted by the exponential function [57]

$$\mathrm{PPF}/\mathrm{PPD}=1+C_{1}e^{-\Delta t/\tau_{1}}+C_{2}e^{-\Delta t/\tau_{2}}$$ (1)

where *C*_1_ and *C*_2_ are facilitation constants and *τ*_1_ and *τ*_2_ represent short-term and long-term relaxation time, respectively. *Δt* is the interval time between 2 consecutive optical spikes. The extracted *τ*_1_ and *τ*_2_ values of PPF/PPD are 70 ms/21 ms and 3.16 s/0.860 s, respectively. Besides the short-term plasticity, the long-term potentiation/depression behaviors can also be modulated with the pulse frequency (Fig. S10). The above results indicate that our all-optically controlled device exhibits evident temporal correlation, which is beneficial for high-efficient image processing.

The heterojunction device has also shown excellent sensitivity to irradiation duration and intensity, which enables real-time processing of complicated image information. In this section, the device current is measured after the UV/visible light is removed for 100 s. As shown in Fig. 3G and H, the device current can be modulated precisely with visible/UV duration and intensity. The long-term potentiation/depression behaviors of 0.756/0.073 nA can be achieved when the irradiation duration increases from 5 to 25 s. Similar potentiation and depression trend can be obtained by applying optical spikes with higher intensity. Furthermore, reversible conductance modulation has also been implemented by utilizing alternate 30 visible and UV spikes. The linearity (determination *R*^2^ in linear fits) of long-term potentiation and depression behaviors is evaluated as 0.886 and 0.995, respectively. The quasi-linear and symmetric conductance evolution is of great significance for high-precision image recognition and classification in artificial neural network.

**Fig. 4.**
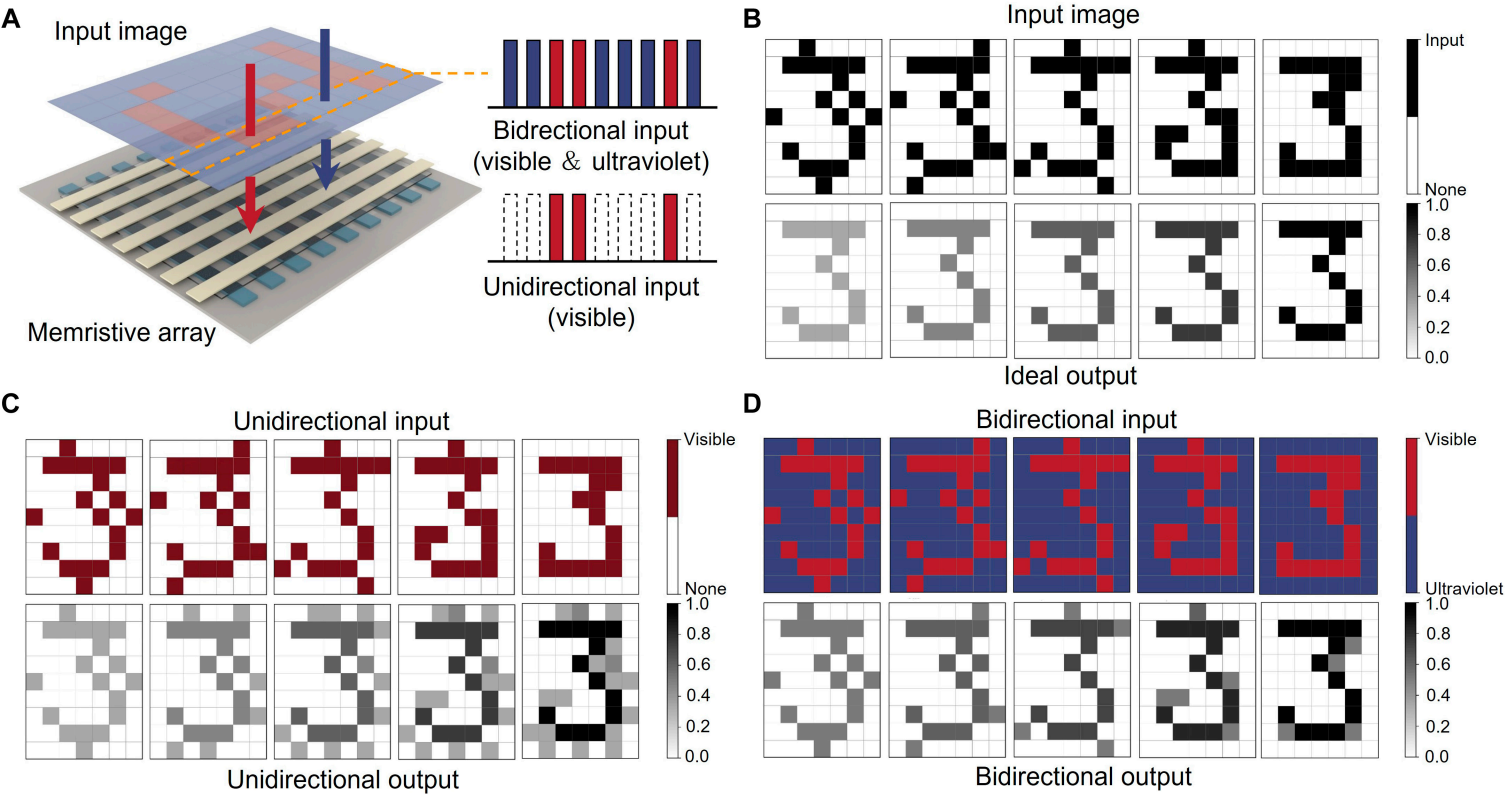
Image preprocessing with all-optical modulation characteristics. (A) Schematic diagram of image sensing in 7 × 9 array. (B to D) Input image with unidirectional (visible) light and bidirectional (visible and UV) light. The bottom panels demonstrate the output image with normalized gray level.

Image preprocessing of biological retina is a critical capability to enhance feature information and suppress random noise, which promotes subsequent high-level image processing in visual cortex [58,59]. In order to emulate the preprocessing function in biological retina, we fabricated a 7 × 9 optoelectronic memristive array (Fig. 4A). The corresponding statistical result of initial current and EPSC values is plotted in Fig. S11. The negligible current fluctuation indicates satisfactory device uniformity. Each memristive unit in the array corresponds to an image pixel. The gray level (from 0 to 255) of each pixel represents the maximum and minimum device current in the array. As shown in Fig. 4B, random noise has been introduced to the ideal image, emulating the inevitable influence of complicated environment. For the unidirectional input, the digital pattern of “3” is irradiated with visible light, while the background represents the dark state. As shown in Fig. 4C, 5 different input images with random noise are sequentially written into the optoelectronic memristor array. The output result of each pixel was recorded after optical input is paused for 100 s. Corresponding output images are plotted in the bottom panel of Fig. 4C. The output image with unidirectional input exhibits pattern “3” with non-negligible image noise. In contrast, we have also performed the image sensing and preprocessing function with bidirectional input (Fig. 4D). For the bidirectional image input, the digital pattern of “3” was written with visible spikes and the background was irradiated with UV light. It is worth noting that the random noise can be effectively suppressed by utilizing UV light in the background. The criteria of structural similarity (SSIM) have been employed to evaluate the noise suppression efficiency (see more details in Materials and Methods). A higher SSIM between preprocessing and ideal image represents better noise suppression process. The SSIM of bidirectional and unidirectional input is 0.8425 and 0.4431, respectively. The output image with bidirectional input has high degree of similarity to the ideal input. As above, our optoelectronic memristive array with bidirectional optical modulation has demonstrated hardware implementation of real-time image preprocessing, which improves the image qualities obtained in non-ideal surroundings.

**Fig. 5.**
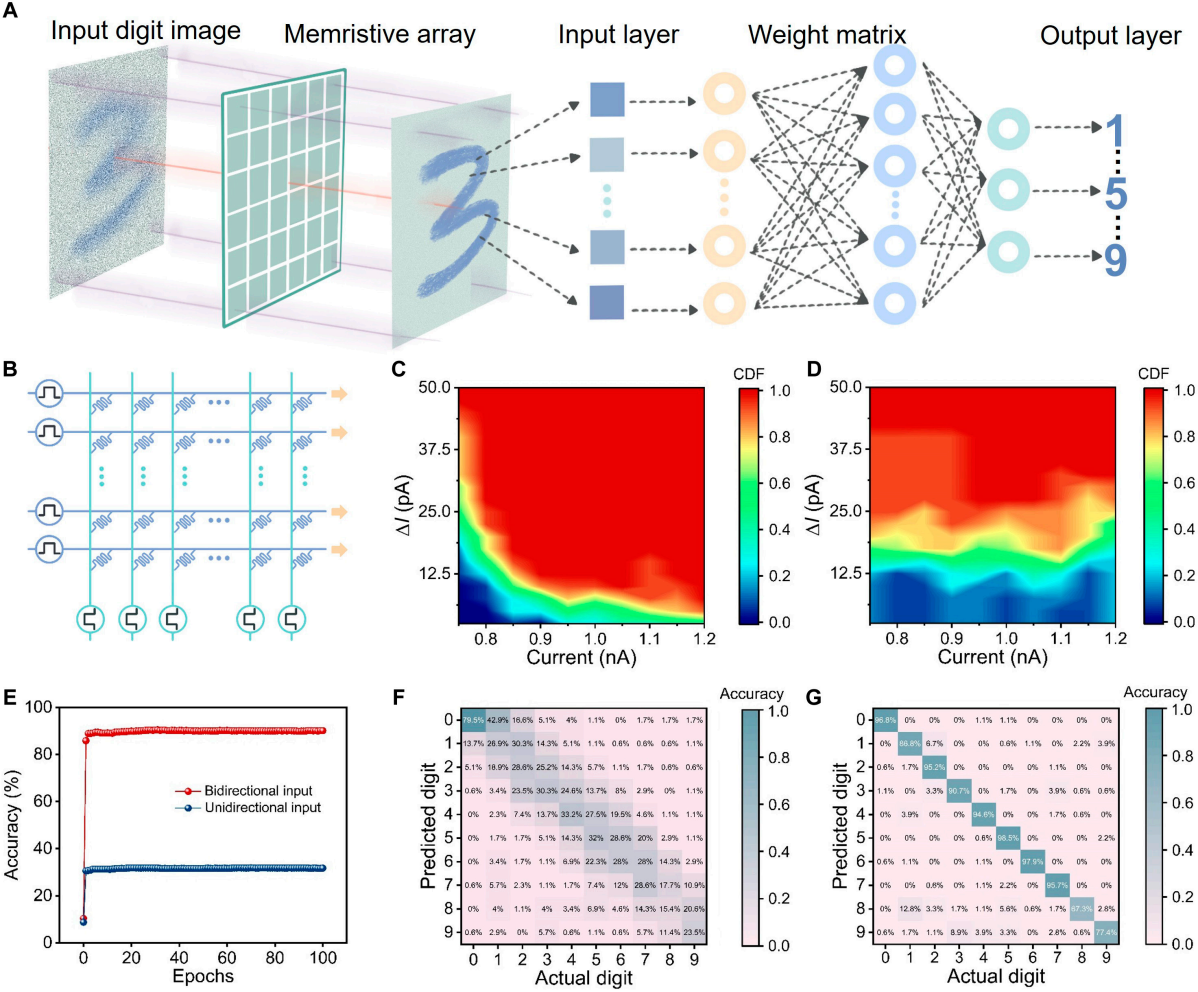
Neuromorphic visual system based on Cu_2_O–TiO_2_-based optoelectronic memristor. (A) Schematic illustration of simulated neuromorphic visual system for handwritten digits. (B) Schematic diagram of conductance evolution in optoelectronic memristive crossbar array. (C and D) CDF of conductance change dependent on device conductance state. (E) Recognition accuracy as the function of training epochs. (F and G) Confusion matrix of classification results for unidirectional and bidirectional input.

Besides the image preprocessing, high-level image processing is also highly required in neuromorphic visual system, including pattern recognition and object classification [60]. In this section, we developed an artificial neural network to perform the classification of handwritten digits using the Modified National Institute of Standards and Technology (MNIST) dataset. As shown in Fig. 5A, the artificial neural network is composed of 784 input neurons, 300 hidden neurons, and 10 output neurons. The schematic diagram of memristive crossbar array is demonstrated in Fig. 5B. The synaptic weight is updated by utilizing the backpropagation (BP) algorithm, which follows the long-term potentiation/depression behaviors in Fig. 3I. Figure 5C and D demonstrates the cumulative distribution function (CDF) of current variation at different current level for potentiation and depression behaviors. The uniform current variation indicates excellent linearity of conductance evolution in our heterojunction device. Then, we prepare training dataset of handwritten digital images with 28 × 28 pixel to train the artificial neural network. As shown in Fig. 5E, the recognition rates of our artificial neural network exceeded 90.0% after 10 training epochs. In contrast, the recognition accuracy without bidirectional preprocessing stabilizes at 31.7% after training. The classification output of handwritten digits is displayed in confusion matrix of Fig. 5F and G. The statistics values in matrix diagonals represent the correct classification of each digit, in which the predicted label is consistent with true label. In can be seen that only 178 digits among the 1797 total samples are mismatched, indicating the high-accuracy classification capability of our all-optically controlled memristor. On the other side, the classification error improves significantly when the bidirectional preprocessing is absent, in which 1197 of 1797 digits (from “0” to “9”) have been classified in error. The quantitative analysis demonstrates that image noise in unidirectional input has a strong impact on classification accuracy. As above, the proposed all-optically controlled device provides an ideal platform to improve imaging qualities for high-performance neuromorphic visual perception.

## Conclusion

In this work, we developed an all-optically controlled synaptic device with the structure of Au/Cu_2_O/TiO_2_/sodium alginate/indium tin oxide. Optical potentiation and depression behaviors can be performed in the proposed device by utilizing visible (680 nm) and UV (350 nm) stimulus, respectively. Several basic synaptic functions have been emulated with optical signals, including excitatory/inhibitory postsynaptic current and paired pulse facilitation and depression. Besides that, our device exhibits the negligible current fluctuation and stable photoresponse behaviors for ~300 d in atmosphere environment. Furthermore, image preprocessing function has been implemented by using the reversible all-optical modulation characteristics. Compared with the unidirectional signals, the bidirectional input with visible and UV light enables to suppress image noise and enhance feature information. On this basis, the accuracy of handwritten digit classification has exceeded 90% in our simulated artificial neural network.

## Materials and Methods

### Device fabrication

All-optically controlled memristor based on Cu_2_O/TiO_2_/sodium alginate nanocomposite was fabricated on the ITO substrate. First, the Cu_2_O nanoparticles are synthesized by dissolving CuCl_2_·2H_2_O (0.852 g) and NaOH (1.20 g) in deionized water and stirring for 10 min at room temperature. Then, l-ascorbic acid (1.760 g) was added into the above solution. The final product was washed with ethyl alcohol and dried in vacuum environment. The Cu_2_O/TiO_2_ dispersion liquid was prepared by adding Cu_2_O nanoparticles (0.03 g), TiO_2_ nanoparticles (0.03 g), and sodium alginate in deionized water and stirring for 10 h. The mass fraction of TiO_2_, Cu_2_O, and sodium alginate is 13.3, 20.0, and 66.7 wt %, respectively. The Cu_2_O–TiO_2_/sodium alginate nanocomposite film was fabricated with the spin-coating method. Finally, the top Au electrodes of 500 μm were deposited by sputtering. SSIM can be expressed as follows [61]:

SSIM(x,y)=f(l(x,y),c(x,y),s(x,y)) (2)

where *l*(*x*, *y*), *c*(*x*, *y*), and *s*(*x*, *y*) represent the image brightness, contrast, and structure, respectively.

### Measurement and characterization

The photocurrent change is monitored by using a semiconductor parameter analyzer (Keithley 2636A) and probe station (TTPX, Lake Shore). Optical signals were performed with a xenon lamp (LA-410UV, Hayashi). All the measurements were performed in ambient atmosphere and room temperature.

## Data Availability

The data used to support the findings of this study are available from the corresponding authors upon request.
