# Supplementary material for: All-Optically Controlled Memristive Device Based on Cu2O/TiO2 Heterostructure Toward Neuromorphic Visual System
Source: Research (Wash D C). 2025 Jan 10;8:0580. doi: 10.34133/research.0580 (PMC11717997; doi:10.34133/research.0580)
Supplement: Supplementary 1 — Figs. S1 to S11 Table S1 [file research.0580.f1.pdf]

Supplementary Materials

All-optically Controlled Memristive Device Based On Cu_2O/TiO_2 Heterostructure Toward Neuromorphic Visual System

Jun Xie, Xuanyu Shan*, Ningbo Zou, Ya Lin*, Zhongqiang Wang*, Ye Tao, Xiaoning Zhao, Haiyang Xu, and Yichun Liu

Key laboratory for UV Light-Emitting Materials and technology (Ministry of Education), College of Physics, Northeast Normal University, China

Table S1 statistical results of all-optical controlled memrsitive device.

Memristive materials	Optical wavelength	Photocurrent	Ref.
NiO/TiO_2	320 nm/480 nm	~0.31 nA	1
PbS/Gr/Pyr-GDY	450 nm/980 nm	~10 μA	2
WO_x/WSe_2	648 nm/375 nm	~70 pA	3
Perovskite/ZnO	365 nm/520 nm	~120 nA	4
P(VDF-TrFE)/CuPc	660 nm/405 nm	~5.1 μA	5
Bi_2O_2Se/graphene	635 nm/365 nm	~2 μA	6
O_D-/O_R-IGZO	420 nm/800 nm	~9 nA	7
$PdSe_2$	1064 nm/473 nm	~14 nA	8
ZnO/PbS/ZnO	365 nm/980 nm	~1 nA	9
Cu_2O-TiO_2/SA	350 nm/570 nm	~17 pA	This work

Reference

[1] Lu et al. Nano Letters (2024): 24, 1667−1672.

[2] Hou et al. ACS nano 15.1 (2020): 1497-1508.

[3] Wu et al. Advanced Functional Materials 33.46 (2023): 2305677.

[4] Ge et al. Advanced Optical Materials 10.11 (2022): 2200409.

[5] Ji et al. Advanced Electronic Materials 8.7 (2022): 2101402.

[6] Yang et al. Advanced Functional Materials 30.30 (2020): 2001598.

[7] Hu et al. Advanced Functional Materials 31.4 (2021): 2005582.

[8] Jiang et al. Small 20.13 (2024): 2306068.

[9] Li et al. Nano Energy 65 (2019): 104000.

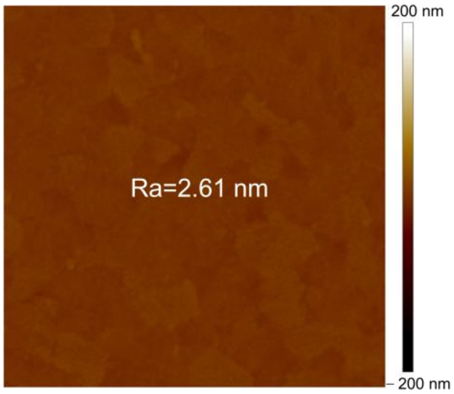

Figure S1. AFM topography image of the Cu_2O-TiO_2 films

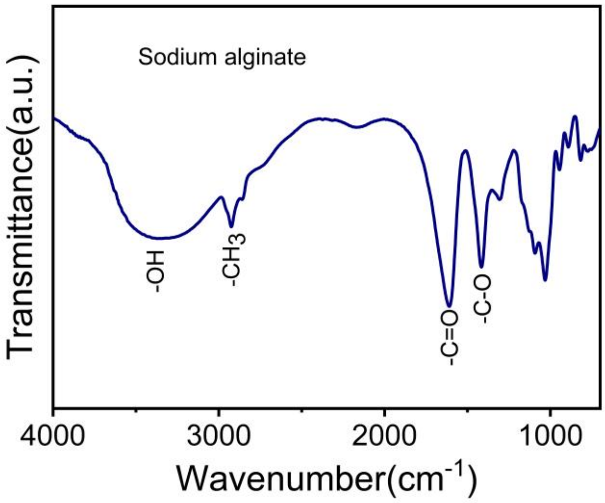

Figure S2. The Fourier transform infrared spectroscopy spectra of sodium alginate.

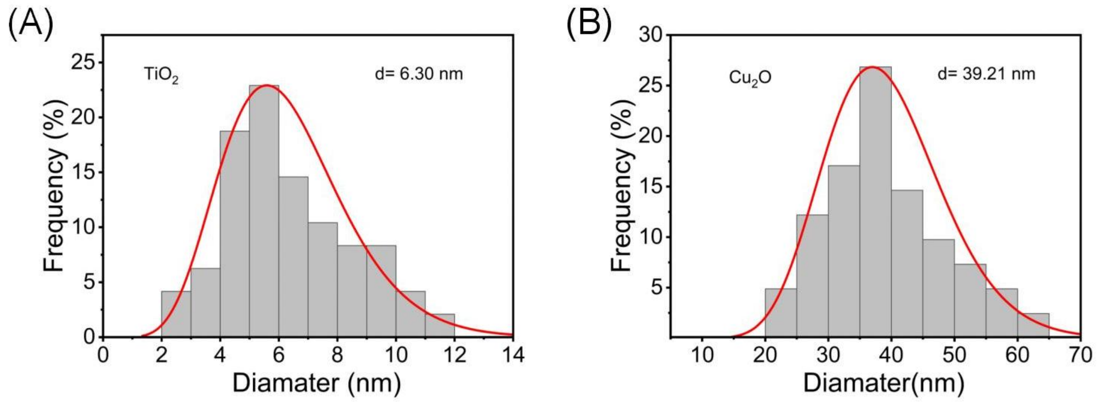

Figure S3. The statistical size distribution of TiO_2 and Cu_2O nanoparticles. The average sizes of TiO_2 and Cu_2O are 6.30 and 39.21 nm, respectively.

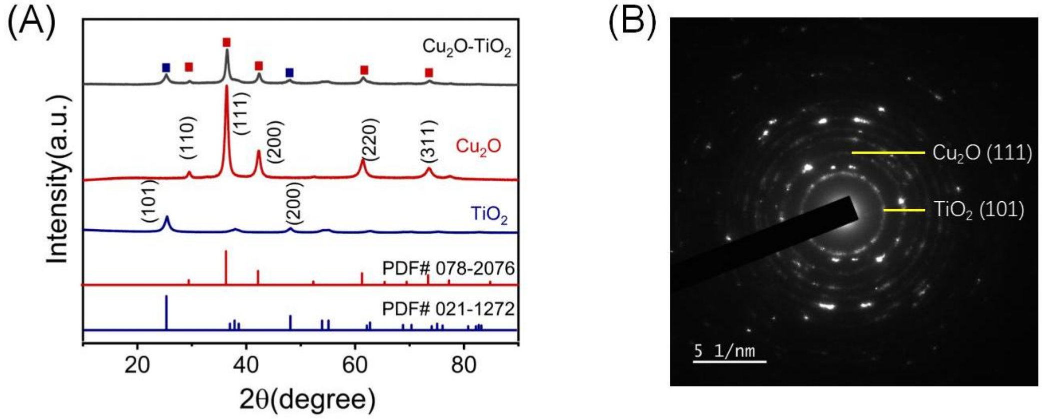

Figure S4. (A) Selected-area electron diffraction (SAED) image of Cu_2O - TiO_2 nanoparticles. The scale bar for the SAED image is 5 nm^{-1}. (B) X-ray diffraction of Cu_2O-TiO_2, Cu_2O and TiO_2 nanoparticles.

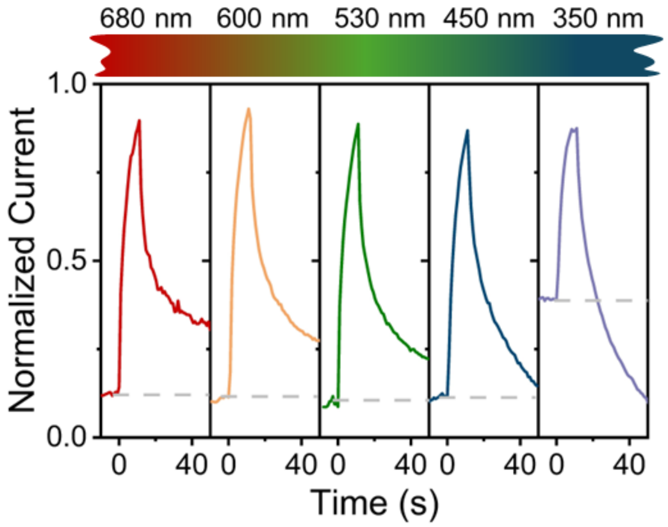

Figure S5. Multiwavelength response of Cu_2O-TiO_2 based device under red (680 nm), yellow (600 nm), green (530 nm), blue (450 nm) and ultraviolet (350 nm) light.

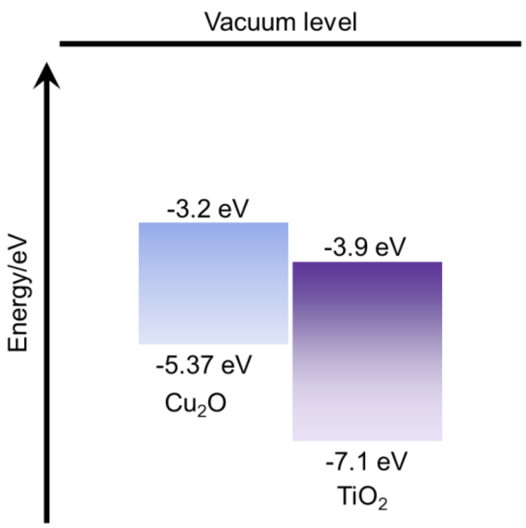

Figure S6. Schematic diagram of the energy band structure of Cu_2O and TiO_2.

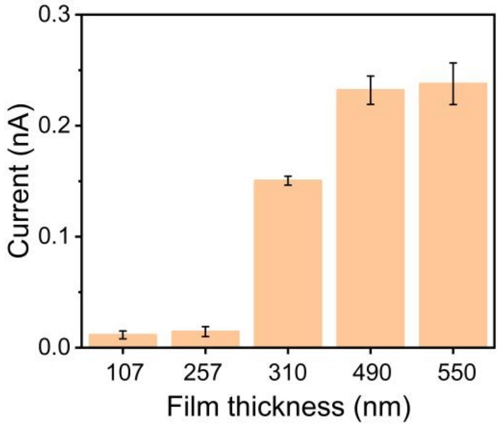

Figure S7. The EPSC amplitude dependent on film thickness. The optical intensity is 17.25 mW/cm^2, the duration is 10 s.

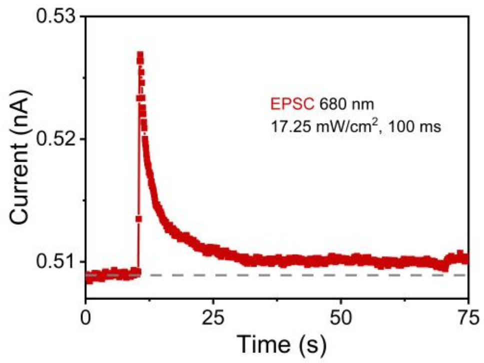

Figure S8. The photoresponse curve under the visible irradiation of 100 ms.

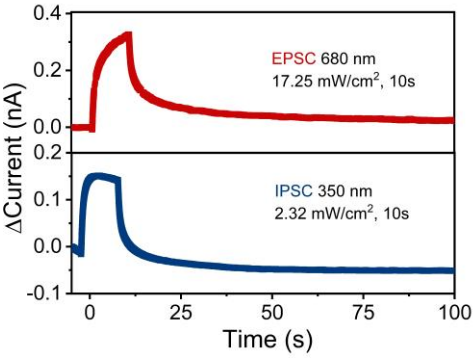

Figure S9. The photoresponse behaviors after placing in atmosphere environment for ~300 days.

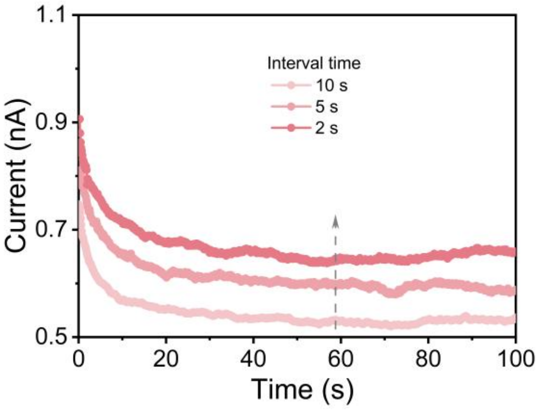

Figure S10. The long-term potentiation behaviors dependent on pulse frequency.

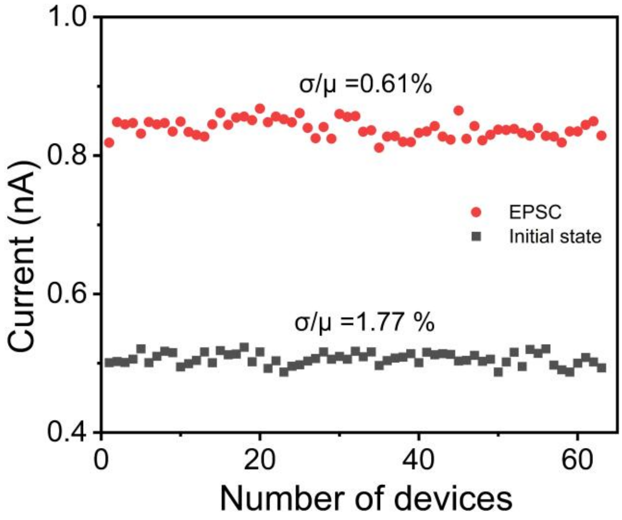

Figure S11. The statistical result of initial and EPSC current.
